# Regulating Rumination by Anger: Evidence for the Mutual Promotion and Counteraction (MPMC) Theory of Emotionality

**DOI:** 10.3389/fpsyg.2017.01871

**Published:** 2017-12-01

**Authors:** Jun Zhan, Fan Tang, Mei He, Jin Fan, Jing Xiao, Chang Liu, Jing Luo

**Affiliations:** ^1^School of Psychology, Capital Normal University, Beijing, China; ^2^School of Marxism, Fujian Agriculture and Forestry University, Fuzhou, China; ^3^School of Labor and Human Resources, Renmin University of China, Beijing, China; ^4^Department of Psychology, The City University of New York, New York City, NY, United States; ^5^School of Psychology, Nanjing Normal University, Nanjing, China

**Keywords:** rumination, anger, sadness, tension, mood induction, MPMC theory of emotionality, traditional Chinese medicine

## Abstract

Unlike the strategy of cognitive regulation that relies heavily on the top-down control function of the prefrontal cortex (PFC), which was recently found may be critically impaired in stressful situations, traditional Chinese philosophy and medicine views different types of emotionality as having mutual promotion and counteraction (MPMC) relationships, implying a novel approach that requires less cognition to emotional regulation. Actually, our previous studies have indicated that anger responses could be successfully regulated via the induction of sadness, and this efficiency could not be influenced by stress, thus providing evidences for the hypothesis of “sadness counteracts anger” (SCA) proposed by the MPMC theory of emotionality (Zhan et al., 2015, 2017). In this study, we experimentally examined the MPMC hypothesis that “anger counteracts rumination” (ACR) which postulates that rumination may be alleviated by the anger emotion. In Study 1, all participants were initially caused state rumination and then induced anger, joy or neutral mood, the results showed that the rumination-related affect was alleviated after anger induction relative to that after joy or neutral mood induction. In Study 2, female participants with high trait rumination were recruited and divided into two groups for exposure to an anger or neutral emotion intervention, the result indicated that the anger intervention group exhibited a greater decline in trait rumination than the neutral emotion intervention group. These findings provided preliminary evidence supporting the hypothesis of ACR, which suggested a new strategy that employs less cognitive resources to regulating state and trait rumination by inducing anger.

## Introduction

**FIGURE 1:**
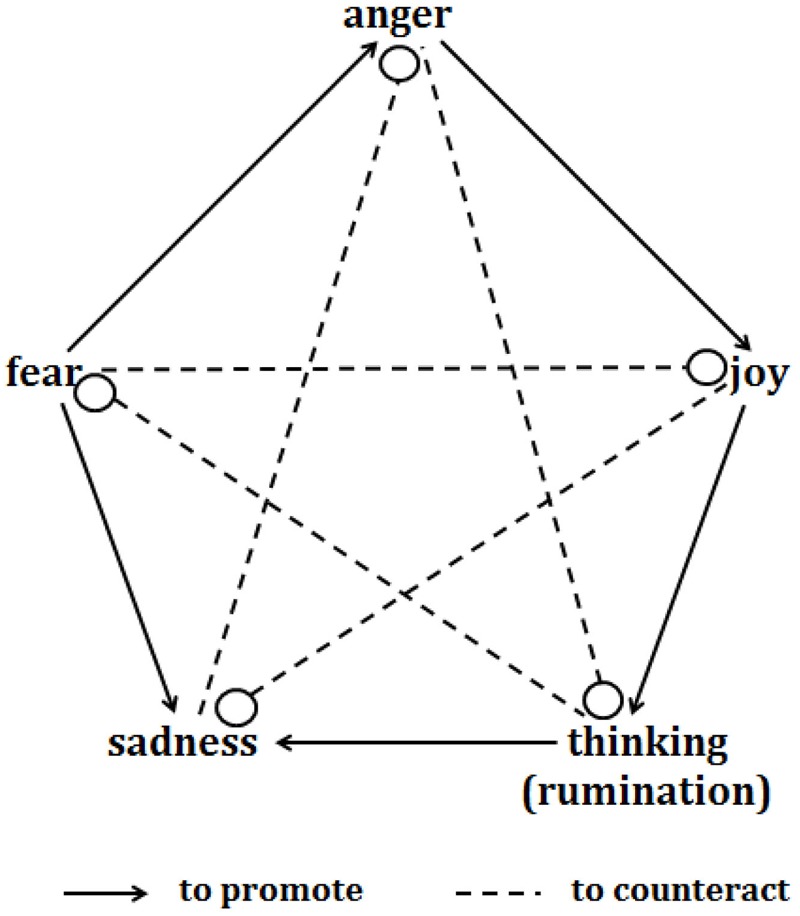
Relationships between mutual promotion and mutual restraint and the emotions of joy, anger, sadness, thinking (which may reflect ordinary thinking and reasoning or rumination) and fear.

As one of the most commonly adopted emotional regulation strategies, cognitive reappraisal has been proven to fail in the regulation of negative emotion under stress. It has been speculated that stress neuroendocrine hormones may impair the executive function of the prefrontal cortex (PFC), thereby undermining cognitive regulation, which depends on the top-down processing of the PFC (Raio et al., 2013). Therefore, emotional regulation strategies that are less reliant on PFC function may be needed for the regulation of negative emotional arousal, particularly the regulation that often occurs under stress. In contrast to the cognitive regulation that emphasizes the role of cognition in executing the top-down regulation of emotion, the MPMC theory of emotionality, which is derived from traditional Chinese medicine, proposes that there are mutual promotion and counteraction (allelopathy) relationships among different types of emotionality, including anger, joy, thinking (rumination)^1^, sadness, and fear. The promotion relationships include “joy promotes thinking,” “thinking (rumination) promotes sadness,” “sadness promotes fear,” “fear promotes anger,” and “anger promotes joy”; the restraint relationships include “joy counteracts sadness,” “sadness counteracts anger,” “anger counteracts thinking (rumination),” “thinking counteracts fear,” and “fear counteracts joy” (**Figure 1**). This theory was first recorded in the “Inner Canon of the Yellow Emperor,” which is the most important ancient text in Chinese medicine that has been treated as the fundamental doctrinal source for Chinese medicine for more than two millennia, basing on the long-term observation of life phenomenon in ancient China, extensive clinical practice and simple anatomical knowledge (Wang, 1999). The MPMC hypothesis proposes a novel approach to emotional regulation, suggesting that one type of emotionality may be regulated by another type, and the relationships of regulating and being-regulated among different emotionalities are specific (e.g., anger may be more efficiently alleviated by sadness than by other emotions). Regarding to the MPMC theory of emotionality, there were two points should be noted: firstly, as we mentioned above (page1, footnote), the original word for “thinking” in the Chinese literature is 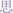 [read as “si”]. 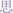 (si, thinking) means either the pure cognitive thinking and reasoning process that is non-pathogenic, or the maladaptive repetitive thinking or ruminative thinking that is typically associated with negative emotion and has pathogenic potentials. Thus, in MPMC theory, the “thinking” (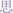
si), may have two different meanings. The “thinking” in the “joy promotes thinking” refers to pure cognitive thinking and reasoning process, and “joy promotes thinking” means the joy emotion may make one to become an active thinker; whereas the “thinking” in the “thinking promotes sadness” or “anger promotes thinking” refers to the maladaptive repetitive thinking and rumination. Therefore, the recursive relationships among joy, thinking and anger is not suitable. Secondly, the figure illustration of MPMC theory of emotionality is a quantitative theoretical assumption in ancient china, this theoretical assumption has not been scientifically tested (the only exception is our recent studies have proved the “sadness counteracts anger” hypothesis made by MPMC theory by well-controlled psychological experiments, Zhan et al., 2015, 2017). The MPMC model of emotionality is based on the mutual promotion and restraint between the five world elements, the *metal, wood, water, fire, and earth*, which were believed to be fundamental by ancient Chinese to constitute the universe and widely used in traditional Chinese medicine to construct its theory to explain various physiological and pathological phenomena. For example, sadness emotion corresponds to the “metal” element, anger emotion corresponds to the “wood” element, and MPMC relationship of “sadness counteract anger” were implied by the principle that wood can be cut by metal (such as ax).

More importantly, this MPMC approach implies an emotional regulation strategy, in which the implementation relies substantially less on the PFC’s cognitive top-control function. Our previous study investigated two hypotheses regarding the modulation of anger from the perspective of the MPMC theory of emotionality: “sadness counteracts (or alleviates) anger” and “fear promotes (or reinforces) anger.” In that study, all of the participants were initially provoked by a designed experimental procedure and subsequently divided into three groups for the induction of sad, fearful, or neutral moods. As predicted, the participants exhibited less anger-related aggression if sadness was evoked later; they reported a higher level of anger if fear was elicited later (Zhan et al., 2015). From the perspective of emotion science, the mechanisms of “sadness counteracts (or alleviates) anger” and “fear promotes (or reinforces) anger” may be interpreted by the interaction between anger and fear and the interaction between anger and sadness. Fear is similar to anger in the neural networks for processing threat signals to the individual (Cannon, 1915; Wager et al., 2015). In contrast, sadness is apparently different from anger in its emotional and neural activation patterns, which prioritize the processing of interoceptive and homeostatic events (Wager et al., 2015). Therefore, there is a strong possibility that the neural activation circuits that support anger and related aggression may be more efficiently cleared by the neural activity underlying sadness.

This study focused on another valuable hypothesis proposed by the MPMC theory of emotionality: “anger counteracts (or alleviates) thinking (rumination).” This hypothesis suggests that anger induction may effectively alleviate ruminative thinking and related negative emotion. A famous case of this hypothesis has been recorded in the literature: in the Warring States Period, a King, Qihuangong, suffered from a disease caused by rumination-like symptoms; a famous doctor named Wenzhi diagnosed his illness. To implement the anger-induction treatment, Wenzhi intentionally broke three appointments with the King, subsequently jumped on the King’s bed without taking off his shoes and insulted him with coarse, provocative words. The King fell into a rage and yelled at Wenzhi. Amazingly, the King’s pre-existing symptoms then resolved (Lv and Bi, 2002). On the basis of the MPMC emotional theory, anger inducing is able to reduce the level of rumination, which could be attributed to the emotional differences between rumination and anger. Rumination is considered to be the result of overthinking, causing fatigue, lethargy and inability to concentrate, while anger was an intense and impulsive emotional response that are always external and without thinking (Li et al., 2007; Wang, 2007). Thus, similar to “sadness counteracts (or alleviates) anger,” the mechanisms of “anger counteracts (or alleviates) rumination” may also be understood from the interaction between anger and rumination. Studies have demonstrated considerable differences in the psychological features and neural networks of anger and rumination. In general, anger is featured by an external and impulsive emotional response tendency that is associated with the approach motivation, whereas rumination is an extreme self-focused state that involves excessive default mode network (DMN) activation (Nolan et al., 1998; Trapnell and Campbell, 1999; Antonucci et al., 2006; Boes et al., 2008; Spreng et al., 2009; Hamilton et al., 2011; Gavita et al., 2012; Dambacher et al., 2015). Therefore, the arousal of anger over rumination may decrease an individual’s ruminative thinking and related negative mood.

As previously discussed, rumination is a negative and maladaptive coping style characterized by prolonged dysphoric reactions to and negatively biased interpretations of problems, potentially maintaining and exacerbating dysphoric affect (Lyubomirsky and Nolen-Hoeksema, 1995). Researchers have suggested that normal rumination or reflective pondering was adaptive to alleviate depression and promote problem solving (Martin and Tesser, 1996; Martin et al., 2004); however, most researchers consider rumination as a non-adaptive coping style that repeatedly and negatively self-focuses on negative events and emotion (Conway et al., 2000; McLaughlin and Nolen-Hoeksema, 2011; Zetsche et al., 2012; Watkins and Nolen-Hoeksema, 2014; Roley et al., 2015). Different types of interventions have been developed to prevent rumination and related depression. For example, individuals who habitually ruminate tend to have a strategic attentional bias toward negative information; thus, distraction from the current event has been determined to decrease an individual’s ruminative thinking (Nolen-Hoeksema, 1991; Rusting and Nolen-Hoeksema, 1998; Donaldson et al., 2007; Yoon and Joormann, 2012; Rabasco et al., 2015); cognitive reappraisal that actively seeks alternate interpretations of the meaning or self-relevance of a negative event has also been demonstrated to be efficient in decreasing rumination (Ray et al., 2008; Keng et al., 2016); moreover, studies of mindfulness-based cognitive therapy (MBCT) have indicated that several weeks of training may help break an individual’s ruminative thinking and sensitivity to negative events, preventing a relapse of depression (Kingston et al., 2007; Segal et al., 2012); besides, expressive writing that requires individuals to write about emotionally upsetting experiences may help individuals reappraise negative events and release emotion and thereby decrease rumination and depressive symptoms (Gortner et al., 2006; Sloan et al., 2008). In contrast to these strategies, the “anger counteracts rumination” (ACR) hypothesis proposes a novel approach to regulate rumination via anger induction. More importantly, relative to the cognitive regulation strategy, the implementation of this approach will require apparently less components of cognitive control that may be vulnerable to stress; in addition, in contrast to expressive writing and meditation, which typically require substantial individual efforts, complicated procedures and relatively long-term training, the implementation of the “ACR” approach is relatively direct and simple.

To address the assumption of “anger counteracts (or alleviates) rumination,” we conducted two experiments to examine whether anger induction may relieve rumination-related thoughts, emotions and personal traits. The first experiment focused on the regulation of state rumination caused by rumination-inducing autobiographical events and contrasted the regulatory effects of anger, joy, and neutral emotions on the cognitive/mnemonic and emotional aspects of rumination. The second study extends the findings of the first experiment to investigate whether the relatively stable trait rumination may be changed by an anger-induction intervention procedure. We conducted a relatively long-term (4 days) anger intervention program that consisted of continuous interventions (one time per day) to examine whether female participants’ trait rumination may be alleviated by an anger intervention.

## Study 1

### Participants

Prior to the formal experiments, we recruited 520 college students from universities in Beijing to complete the ruminative response scale (RRS). The RRS is the most widely used instrument to measure rumination. It includes 22 items rated on a four-point scale (1-almost never, 2-sometimes, 3-often, and 4-almost always) and addresses how often participants engage in responses to feeling sad or depressed (Nolen-Hoeksema and Morrow, 1991; Han and Yang, 2009). 105 participants with high scores (*M* = 62.70, *SD* = 5.15) on the RRS (approximately top 20% among 520 college students) were selected to participate in the formal experiments. The reason we selected subjects with high RRS scores was to make the success of rumination induction, because it could be very hard to induce subjects with low RRS scores to fall into rumination state by a simple and brief experimental task. The data from 15 participants were excluded from the final analysis because 11 participants stopped participating in the formal experiments, 3 participants correctly guessed the experimental purpose, and 1 participant had missing data as a result of a technical problem with e-prime. The remaining 90 participants (age: *M* = 22, *SD* = 1.34) were randomly assigned to anger group (female: *n* = 13, male: *n* = 17), joy group (female: *n* = 19, male: *n* = 11) or neutral group (female: *n* = 18, male: *n* = 12), with each group consisting of 30 participants. This study was carried out in accordance with the recommendations of ‘The ethical rules of psychological experiment of human subjects, Capital Normal University’s Committee’ with written informed consent. All of the participants signed the informed consent form and each participant was compensated 30 RMB for study participation.

### Experimental Design and Procedures

#### Overview of Experimental Procedure

**FIGURE 2:**
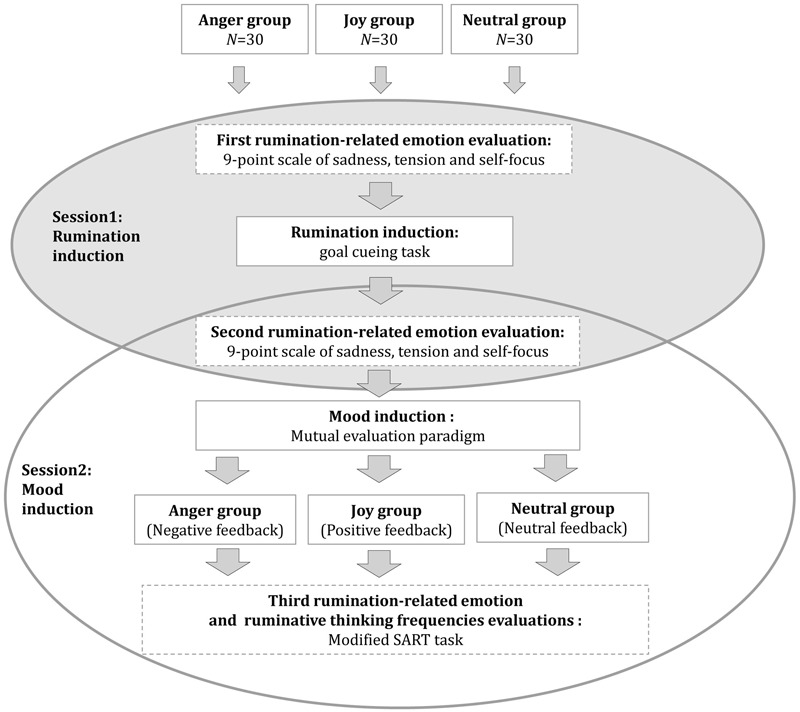
Schematic illustration of the experimental procedure in Study 1.

To test our predictions, a single-factor (group: anger group, joy group, neutral mood group) between-group design was used in the present study. The entire experimental procedure consisted of two stages (**Figure 2**). The first is the rumination induction phase, the participants were instructed to identify an unresolved event from the preceding week about which he/she repeatedly experienced concern (rumination event); they were required to freely recall the event, keep it in mind, and write down the related contents. This phase lasted approximately 10 min. Participants were required to rate the levels of rumination-related emotions (sadness, tension, and self-focus) before (at baseline) and immediately after this period. The second is the mood induction stage, the participant was randomly assigned to an anger group, a joy group or a neutral mood group (with the restriction of maintaining a rough balance with respect to the sex ratio in each group) to be subjected to the corresponding emotion evoking procedure. At the end of this period, the participant was required to complete the “sustained attention to response task” (SART) irregularly inserted with probing questions to evaluate state rumination-related emotions (sadness, tension, and self-focus), along with the frequencies of ruminative thought (attention degrees on the rumination event recalled in the first period).

#### Evaluation of Positive and Negative Emotion

The Positive and Negative Affect Schedule (PANAS, Watson and Tellegen, 1985) was used to assess the participants’ emotional states at baseline, after rumination induction and immediately after the mood induction. This scale was used and confirmed with its applicability in Chinese version (Huang et al., 2003). Positive affect was measured in using 10 adjectives from the positive affect subscale of the PANAS; while negative affect was measured in using 10 adjectives from the negative affect subscale of the PANAS. All of the adjectives were rated along a 5-point Likert-type scale: 1 (very slightly or not at all), 2 (a little), 3 (moderately), 4 (quite a bit), and 5 (extremely). At the beginning of the session, participants were told to “Indicate the extent to which you feel this way right now, that is, at the present moment.” The scores for the adjectives of the positive affect subscale and negative affect subscale were added to obtain the levels of positive and negative emotions, respectively.

#### Evaluation of Subjective Anger Feeling

The subjective anger feeling was evaluated at baseline, after rumination induction and immediately after the mood induction, which was measured by the hostility subscale of the revised Multiple Affect Adjective Checklist (MAACL) (Zuckerman and Lubin, 1985). According to the method reported in a related experiment performed by Bushman et al. (2001). In the Chinese version of the MAACL (Zhang, 1991), the hostility subscale contains 22 adjectives, including 11 words that are positively associated with anger (irritable, cruel, jealous, disgruntled, indignant, impatient, hostile, irritated, violent, furious, and exasperated) and 11 words that are negatively associated with anger (gracious, easy-going, good-natured, helpful, friendly, courteous, gentle, pleasantly agreeable, kind, affable, and cooperative). The participants were asked to check these 22 adjectives according to their feelings at that time. When they selected a word that was positively associated with anger or unselected a word that was negatively associated with anger, they accumulated one point; the final scores were the sum of the total points. High total scores indicate a high level of anger.

#### Evaluation of Rumination-Related Emotions

The rumination-related emotions (sadness, tension, and self-focus) were evaluated at baseline, after rumination induction and after the mood induction (being probed in the SART). The participant was instructed to complete the 9-point scale regarding his/her feelings of sadness, tension, and self-focus emotions at the time. The subjects selected from 1 (not at all) to 9 (very) to describe their current levels of sadness, tension and self-focus; high scores indicate a high level of each type of emotion (Roberts et al., 2013; Watkins and Nolen-Hoeksema, 2014).

#### Rumination Induction

The rumination induction procedure was administered after the participants completed the baseline rating. The participants were instructed to identify an ongoing and unresolved concern that had repeatedly come into their mind and caused them to feel negative or stress during the previous week (Behar et al., 2005). Examples of problems were provided (e.g., “If you had trouble with an important friend, would you feel upset because of improper dealing with the relationship between you two?” “If you met something unpleasant, would you be troubled all the time?” “If something awkward happened to you in a momentous occasion, would you feel down all the time?” “If what you did disappointed someone important to you, would you be sad?” “You feel less competitive on something you care about”). The participants were required to briefly outline the problem that they had identified prior to this goal focus period. A 10 min goal focus period followed, during which the participants were asked to focus on the concern they identified and to write about it on a blank piece of paper, which they could choose to take with them after the experiment.

#### Mutual Evaluation Paradigm for Inducing Anger/Joy/Neutral Emotion

The participants were then randomly assigned to one of three groups to induce anger, joy, or neutral emotion using a modified mutual (essays) evaluation paradigm (Bushman et al., 1999, 2001; Bushman, 2002). Each participant was led to believe that he or she would be interacting with another participant, and they were required to write a paragraph that focused on a popular topic in Chinese society (e.g., a Nobel Prize winner commented on the Chinese younger generation’s severe competition civil servant positions) and express their views on the subject. Each participant was told that another participant (who did not actually exist) was completing the questionnaire in another room; they would subsequently evaluate each other’s views using a score that ranged from -10 (very poor) to 10 (very good) and a brief comment. When the participant completed the writing of his/her viewpoint, the experimenter took the comments and claimed that they would be reviewed by the other participant. The experimenter also presented the “other” participant’s paper (prepared in advance by the experimenter) to the participant and asked him/her to carefully read the paper, assign a score, and provide a brief comment. The experimenter subsequently showed the participant the extremely negative, extremely positive or neutral evaluation of his/her viewpoint to induce the participant’s anger, joy, or neutral emotion. Specifically, the participant in the anger group received a score of -10 and an insulting criticism regarding his/her opinion, writing level, attitudes and moral quality, whereas the participant in the joy group received a score of 10 and a favorable review regarding his/her view; the participant in the neutral mood group received a score of 5 and a neutral comment regarding his/her viewpoint.

#### Probing Rumination-Related Thoughts and Emotion in SART

A modified “sustained attention to response task” (SART) was adopted to dynamically and immediately measure the state rumination (ruminative thought frequency and rumination-related negative affect) (Roberts et al., 2013). The SART used a simple go/no-go paradigm to elicit a repetitive automatic style of responding to the stimuli, during which thought probes and mood probes were presented to indicate where the participant’s attention was focused and how they immediately felt (Robertson et al., 1997; Roberts et al., 2013). With reference to Roberts et al. (2013)’s research, the SART presented the participants with 1800 neutral words; each word was presented for 300 ms. Most trials required the participants to respond to a lowercase word with the “p” key button and to withhold their response when the presented word was in uppercase. The task consisted of four blocks, each of which presented 450 trials, including 45 words repeated ten times in a different order; five uppercase words and 40 lowercase words were randomly arranged within each set of 45 words. There was no recognizable break between the four blocks. To investigate state rumination, thought probes of personal goals, including ruminative thinking, were pseudo-randomly probed following the 25th or 50th no-go trials within each block. In contrast to Roberts’s study instructing participants to select from six response options to describe what they had just been thinking about just before the probe, this study required the participants to provide a yes or no response to six questions. These questions were as follows: (1) Did you think of the task? (2) Did you think of performance in the task? (3) Did you think of sleepiness, hunger or other physical conditions? (4) Did you think of worries or concerns identified in the previous written task? (5) Did you think of the peer response comment by the other participant in the peer response task? (6) Did you think of other types of thoughts? The “yes” frequency of the fourth question was regarded as the index of state ruminative thinking. Participants additionally rated their rumination-related mood following each probe using bipolar computerized scales where they pressed the keys (1–9) that best described their degree of sadness, tension and self-focus, respectively, and the average of each emotion level was used to evaluate corresponding emotion in SART. The entire task lasted 60 min (Robertson et al., 1997; Roberts et al., 2013).

### Results

#### Positive Emotion

**FIGURE 3:**
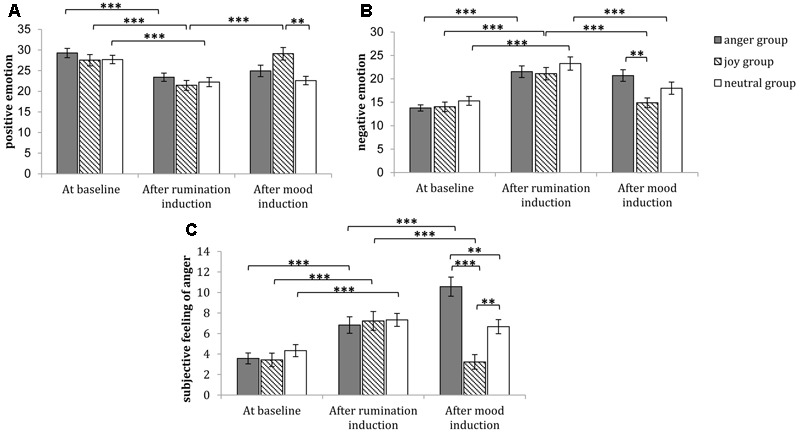
The emotional examination of experimental manipulation in Study 1. The subjective feelings of positive emotion at baseline, after rumination induction and after mood induction are shown in **(A)**. The subjective feelings of negative emotion at baseline, after rumination induction and after mood induction are shown in **(B)**. The subjective feelings of anger at baseline, after rumination induction and after mood induction are shown in **(C)**. And after mood induction, the positive level of joy group was marginally higher than that of anger group (*p* = 0.0084). Error bars (capped vertical bars) represent (–1)/(+1) *SE*. ^∗∗∗^*p* < 0.001, ^∗∗^*p* < 0.01.

The 3 (time: at baseline, after rumination induction, after mood induction) × 3 (group: anger group, joy group, neutral mood group) repeated measures ANOVA of positive emotion indicated that the main effect of time was significant [*F*_(2,174)_ = 48.857, *p* < 0.001, η^2^ = 0.360], the interaction between time and group was significant [*F*_(4,174)_ = 9.441, *p* < 0.001, η^2^ = 0.178]. A simple effect analysis (sidak-adjusted) focusing on the time differences showed that the positive emotion level after rumination induction was significantly lower than that at baseline in all groups (*p*s < 0.001); meanwhile, for joy group, the positive emotion level after mood induction was significantly higher than that after rumination induction (*p* < 0.001), but for anger group and neutral mood group, the positive emotion level after rumination induction had no significant differences with that after mood induction (*p*s > 0.05) (see **Figure 3A**). A simple effect analysis (sidak-adjusted) focusing on the group differences found that the positive emotion level at baseline and that after rumination induction had no significant differences between any two groups (*p*s > 0.05); but after mood induction, the positive level of joy group was significantly or marginally higher than that of neutral mood group (*p* < 0.01) and anger group (*p* = 0.0084) (see **Figure 3A**).

#### Negative Emotion

The 3 (time: at baseline, after rumination induction, after mood induction) × 3 (group: anger group, joy group, neutral mood group) repeated measures ANOVA of negative emotion indicated that the main effect of time was significant [*F*_(2,174)_ = 61.102, *p* < 0.001, η^2^ = 0.413], the interaction between time and group was significant [*F*_(4,174)_ = 4.236, *p* < 0.01, η^2^ = 0.089]. A simple effect analysis (sidak-adjusted) focusing on the time differences showed that the negative emotion level after rumination induction was significantly higher than that at baseline in all groups (*p*s < 0.001); meanwhile, for joy and neutral mood group, the negative emotion level after mood induction was significantly lower than that after rumination induction (*p* < 0.001), but for anger group, the negative emotion level after rumination induction had no significant differences with that after mood induction (*p* > 0.05) (see **Figure 3B**). A simple effect analysis (sidak-adjusted) focusing on the group differences found that the negative emotion level at baseline and that after rumination induction had no significant differences between any two groups (*p*s > 0.05); but after mood induction, the negative level of joy group was significantly lower than that of anger group (*p* < 0.01) (see **Figure 3B**).

#### Subjective Feeling of Anger

The 3 (time: at baseline, after rumination induction, after mood induction) × 3 (group: anger group, joy group, neutral mood group) repeated measures ANOVA of anger feeling indicated that the main effect of time was significant [*F*_(2,174)_ = 45.001, *p* < 0.001, η^2^ = 0.341], the interaction between time and group was significant [*F*_(4,174)_ = 20.765, *p* < 0.001, η^2^ = 0.323]. A simple effect analysis (sidak-adjusted) focusing on the time differences showed that the subjective anger feeling after rumination induction was significantly higher than that at baseline in all groups (*p*s < 0.001); meanwhile, the subjective anger feeling after anger induction was significantly higher than that after rumination induction (*p* < 0.001), the subjective anger feeling after joy induction was significantly lower than that after rumination induction (*p* < 0.001), the subjective anger feeling after rumination induction had no significant differences with that after neutral mood induction (*p*s > 0.05) (see **Figure 3C**). A simple effect analysis (sidak-adjusted) focusing on the group differences found that the subjective anger feeling at baseline and that after rumination induction had no significant differences between any two groups (*p*s > 0.05); but after mood induction, for the subjective anger feeling, the anger group was significantly higher than that of joy group (*p* < 0.001) neutral mood group (*p* < 0.01), and the joy group was significantly lower than that of neutral mood group (*p* < 0.01) (see **Figure 3C**).

#### Subjective Feelings of Sadness

**FIGURE 4:**
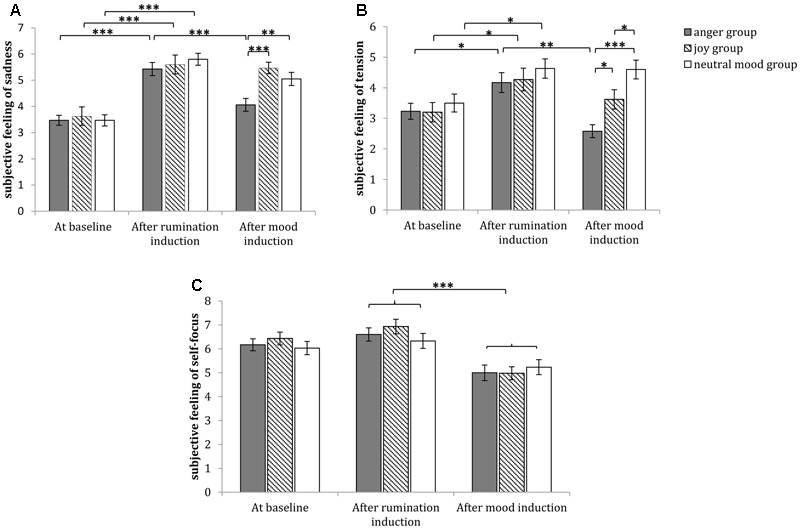
Comparisons of emotional changes in the anger group, joy group and neutral group in Study 1. The subjective feelings of sadness at baseline, after rumination induction and after mood induction are shown in **(A)**. The subjective feelings of tension at baseline, after rumination induction and after mood induction are demonstrated in **(B)**. The subjective feelings of self-focus at baseline, after rumination induction and after mood induction are demonstrated in **(C)**, and the self-focus level after rumination induction was marginally significantly higher than that at baseline (*p* = 0.058). Error bars (capped vertical bars) represent (–1)/(+1) *SE*. ^∗∗∗^*p* < 0.001, ^∗∗^*p* < 0.01, ^∗^*p* < 0.05.

The 3 (time: at baseline, after rumination induction, after mood induction) × 3 (group: anger group, joy group, neutral mood group) repeated measures ANOVA of sadness indicated that the main effect of time was significant [*F*_(2,174)_ = 62.614, *p* < 0.001, η^2^ = 0.419], the main effect of group was marginally significant [*F*_(2,87)_ = 2.847, *p* = 0.063, η^2^ = 0.061], the interaction between time and group was significant [*F*_(4,174)_ = 2.630, *p* < 0.05, η^2^ = 0.057]. A simple effect analysis (sidak-adjusted) focusing on the time differences showed that the sadness level after rumination induction was significantly higher than that at baseline in all groups (*p*s < 0.001); meanwhile, for anger group, the sadness level after mood induction was significantly lower than that after rumination induction (*p* < 0.001), but for joy group and neutral mood group, the sadness level after rumination induction had no significant differences with that after mood induction (*p*s > 0.05) (see **Figure 4A**). A simple effect analysis (sidak-adjusted) focusing on the group differences found that the sadness level at baseline and that after rumination induction had no significant differences between any two groups (*p*s > 0.05); but after mood induction, the sadness level of anger group was significantly lower than that of joy group (*p* < 0.05) and neutral mood group (*p* < 0.001) (see **Figure 4A**).

#### Subjective Feelings of Tension

The 3 (time: at baseline, after rumination induction, after mood induction) × 3 (group: anger group, joy group, neutral mood group) repeated measures ANOVA of tension indicated that the main effect of time was significant [*F*_(2,174)_ = 11.021, *p* < 0.01, η^2^ = 0.112], the main effect of group was significant [*F*_(2,87)_ = 5.286, *p* < 0.001, η^2^ = 0.108], the interaction between time and group was significant [*F*_(4,174)_ = 2.952, *p* < 0.05, η^2^ = 0.064]. A simple effect analysis (sidak-adjusted) focusing on the time differences showed that the tension level after rumination induction was significantly higher than that at baseline in all groups (*p*s < 0.05); meanwhile, for anger group, the tension level after mood induction was significantly lower than that after rumination induction (*p* < 0.01), but for joy group and neutral mood group, the tension level after rumination induction had no significant differences with that after mood induction (*p*s > 0.05) (see **Figure 4B**). A simple effect analysis (sidak-adjusted) focusing on the group differences found that the tension level at baseline and that after rumination induction both had no significant differences between any two groups (*p*s > 0.05); in contrast, after mood induction, the tension level of anger group was significantly lower than that of neutral mood group (*p* < 0.001) and joy group (*p* < 0.05), and the tension level of joy group was significantly lower than that of neutral mood group (*p* < 0.05) (see **Figure 4B**).

#### Subjective Feelings of Self-focus

The 3 (time: at baseline, after rumination induction, after mood induction) × 3 (group: anger group, joy group, neutral mood group) repeated measures ANOVA of self-focus indicated that the main effect of time was significant [*F*_(2,174)_ = 41.642, *p* < 0.001, η^2^ = 0.324], the main effect of group [*F*_(2,87)_ < 1, *p* > 0.05, η^2^ = 0.007] and the interaction between time and group were not significant [*F*_(4,174)_ = 1.089, *p* > 0.05, η^2^ = 0.024]. Regarding to the significant main effect of time, the multiple comparisons (sidak-adjusted) showed that the self-focus level after rumination induction was marginally significantly higher than that at baseline (*p* = 0.058), meanwhile, the self-focus level after mood induction were significantly lower than that after rumination induction and that at baseline (*p*s < 0.001) (**Figure 4C**).

#### Thought Frequency

The one-way ANOVA of the thought frequency of the six probing rumination-related thoughts indicated that there were no significant group differences in the frequency of Q1 (thoughts of the task) [*F*_(2,87)_ < 1, *p* > 0.05, η^2^ = 0.031], Q2 (task performance) [*F*_(2,87)_ < 1, *p* > 0.05, η^2^ = 0.031], Q3 (physical state) [*F*_(2,87)_ < 1, *p* > 0.05, η^2^ = 0.032], Q4 (the goal event or concerns written in the rumination induction step) [*F*_(2,87)_ < 1, *p* > 0.05, η^2^ = 0.044], Q5 (peer review materials) [*F*_(2,87)_ < 1, *p* > 0.05, η^2^ = 0.055], and Q6 (any other thoughts) [*F*_(2,87)_ < 1, *p* > 0.05, η^2^ = 0.058].

### Brief Summary of Study 1

Study 1 investigated the effects of inducing anger, joy, and neutral mood on the previously evoked state rumination and related sadness, tension, and self-focus feelings. First of all, the results indicated tha manipulations of rumination induction and mood induction were efficient, the rumination induction significantly increased the level of rumination-related emotion, negative emotion and decreased the level of positive emotion, the anger induction significantly increased the subjective anger feeling (relative to joy induction and neutral mood induction), the joy induction significantly increased the positive emotion (relative to anger induction and neutral mood induction), moreover, there were no significant group differences in feelings of these emotions before and after rumination induction, providing a suitable basis for conducting the mood induction manipulations. Secondly, consistent with previous research, state rumination-related sadness, tension, and self-focus decreased after mood induction, which may be mainly attributed to a distraction effect (Nolen-Hoeksema, 1991). Notably, the results supported the “anger counteracts (or alleviates) rumination” hypotheses in the finding that the sadness and tension were both significantly decreased after anger induction. What’s more, joy induction (contrast with neutral mood induction) decreased the rumination-related tension, which indicates that positive emotion may also be helpful for relieving rumination-related mood, but its efficiency is inferior to that of anger. However, there were no significant differences in the ruminative thinking frequencies after anger induction, joy or neutral mood induction. These findings indicated that anger induction may target the emotional experience associated with rumination (characterized by the feelings of sadness and tension) but not the aspects of cognition and memory retrieval (characterized by the frequency of state ruminative thoughts in the SART). We also conducted the correlation analysis of ruminative emotion and thinking, and no significant correlation between them was detected (*p*s > 0.05), and the analyzed output of spss has been uploaded as Supplementary Materials (**Supplementary Figure S1**). In our opinion, the inefficient results on ruminative thinking could be mainly attributed to strategy of “anger counteracts rumination” acted on the alteration of rumination-related emotion rather than cognition.

Therefore, Study 1 testified that anger was efficient in alleviating state rumination-related sadness and tension emotions, but it remains unclear whether the more fundamental aspects of rumination (such as trait rumination) may also be relieved by anger induction. To investigate this issue, a relatively long-term intervention of anger induction was implemented in Study 2 to determine whether trait rumination may also be changed by anger treatment in which four types of anger induction were conducted over four continuous days, respectively.

## Study 2

### Participants

In general, women have a more ruminative response style than men (Nolen-Hoeksema, 1991), and they tend to be more sensitive to emotional manipulations; thus, only female college students with relatively high scores on the RRS were recruited for this study (approximately top 10% among 465 participants). Forty female participants from universities in Beijing (RRS: *M* = 47.88, *SD* = 6.50; age: *M* = 22.15, *SD* = 1.578) were selected to participate in the formal experiments; they were randomly assigned to an anger emotion intervention group and a neutral emotion intervention group with 20 participants per group. This study was carried out in accordance with the recommendations of ‘The ethical rules of psychological experiment of human subjects, Capital Normal University’s Committee’ with written informed consent. All of the participants signed the informed consent form and each participant was compensated 150 RMB for study participation.

### Experimental Design and Procedures

We induced four episodes of anger and neutral mood induction for the anger group and neutral group, respectively. With one anger or neutral mood intervention assigned per day, the entire intervention period was conducted over four consecutive days. There is no standardized procedure to evoke anger emotion multiple times in a relatively long-term intervention period; thus, we implemented experimental procedures that have been demonstrated to result in anger emotions, including watching anger-inducing movie clips and social news and participating in the Taylor Aggression Paradigm and Mutual Essay Evaluation Paradigm as the anger-inducing intervention approaches. Corresponding with these anger-inducing procedures, matched experimental procedures that will lead to neutral emotions were adopted to induce the control condition for comparison. Each intervention lasted approximately 13 ∼ 15 min. The trait rumination level was evaluated by the RRS as used in Study 1; and the feelings of anger were measured after each emotion intervention by the hostility subscale of the Multiple Affect Adjective Checklist (MAACL) to examine the anger induction manipulation (Zuckerman and Lubin, 1985). In the Chinese version of MAACL (Zhang, 1991), the subscale includes 11 words that have positive associations with anger and 11 words that have negative associations with anger. The participants were required to check these 22 adjectives according to their feelings at that time. When they selected a word that was positively associated with anger or unselected a word that was negatively associated with anger, they accumulated one point, and the final score was the sum of the total points, a high total score indicates a high level of anger. The details regarding each time (day) of emotion intervention are as follows.

On the 1st day of the emotion-inducing intervention, the participants were required to watch standardized movie clips, which were used to induce anger and neutral moods in the anger and neutral groups, respectively. Both the anger-inducing and neutral emotion-inducing movie clips were obtained from the Chinese Emotional Visual Stimulus (CEVS) database for inducing anger or neutral emotions (Xu et al., 2010). The anger-inducing movie clips included: Video Clips A-1: duration: 2′43″, from the movie “the Tokyo Trial”; Video Clips A-2: duration: 4′17″, from the movie “Fist of Fury”; Video Clips A-3: duration: 1′06″, from the movie “Fist of Fury”; Video Clips A-4: duration: 1′34″, from the movie “Kangxi Dynasty”; and Video Clips A-5: duration: 1′47″, from the movie “Conman in Tokyo.” The neutral emotion-inducing movie clips included: Video Clips N-1: duration: 1′14″, from the movie “Introduce the projector”; Video Clips N-2: duration: 1′10, from the movie “IP package”; Video Clips N-3: duration: 1′08″, from the movie “Introduce the hardware conflict”; Video Clips N-4: duration: 2′02″, from the movie “Computer Repair 1”; Video Clips N-5: duration: 1′11″, from the movie “interfaces fix”; and Video Clips N-6: duration: 6′01″, from the movie “Computer Repair 2.” All of the anger or neutral video clips were continuously played in a random order with a short time interval between the presentations of the video clips. The angry and neutral movies both lasted approximately 13 min. While watching the movies, the participants were required to attempt to concentrate on the movie and experience their natural feelings.

On the 2nd day of the emotion-inducing intervention, the participant was required to participate in the Taylor Aggression Paradigm (TAP), which has been widely used to induce anger and aggressive behaviors (Taylor, 1967; Bushman et al., 1999; Giancola and Parrott, 2008; Dambacher et al., 2015). The TAP was presented as a competitive reaction time task, which was used to induce an angry or neutral mood by providing extremely high or relatively low levels of punishment. In this task, the participant was informed that he/she would be paired with another player, whom he/she did not meet in person. The TAP required the participants to press a button as quickly as possible on each trial of stimulus presentation; the participant who was slower would receive a blast of white noise (similar to radio static) through headphones. Each participant was permitted to set the intensity of the noise that the other individual would receive between 60 decibels (Level 1) and 105 decibels (Level 10) if the other individual lost. All of the noises were produced using Praat speech software (a free scientific computer software package that was designed by Paul Boersma and David Weenink of the University of Amsterdam). In the anger intervention, the “partner” always set a high intensity (from level 5 to level 10, with an average level of 9) throughout the task session; the participant lost 16 times in 20 rounds. In the neutral mood intervention, the “partner” set random noise levels throughout the task session, with half winning rounds and half losing rounds. The TAP process lasted approximately 10 min.

On the 3rd day of the emotion-inducing intervention, the participant was required to watch video clips of negative news or neutral mood clips. Through the Internet, the experimenter collected 12 video clips that reported controversial events that clearly violated the principles of modern morality and humanity and had a strong possibility to evoke anger (Amodio et al., 2007). A random sample of 10 undergraduate students were asked to evaluate their emotional feelings, particularly anger-related feelings, after watching these video clips using 9-point scale. Of the 12 pieces of video clips, five clips with relatively higher evaluation scores of anger were ultimately selected as anger-inducing material for the formal anger-inducing intervention study (mean anger intensity of the 5 video clips: *M* = 7.8, *SD* = 2.571), including clips described as “nanny hit baby” (duration: 1′38″), “wicked son killed his mother” (duration: 1′36″), “second-generation rich swollen with arrogance” (duration: 2′44″), “real estate developers forced demolition of houses” (duration: 5′39″), and “old man pulling bus driver led to several cars colliding” (duration: 2′03″). All video clips of anger or neutral were continuously played in a random order within a short time. While watching the movies, the participants were required to attempt to concentrate on the movie, express their natural feelings and not suppress emotion. The movie clips of the neutral mood were the same as the clips used in intervention 1, which were all selected from the CEVS database (Xu et al., 2010). The video clips of the anger news and neutral mood movie both lasted approximately 13 min.

On the 4th day of the emotion-inducing intervention, the modified Mutual Evaluation Paradigm was conducted to induce anger and neutral moods; the process was the same as Study 1 with the exception that the treatment of the joy mood induction (extremely positive emotional feedback) was not included.

### Results

#### Subjective Feeling of Anger

**FIGURE 5:**
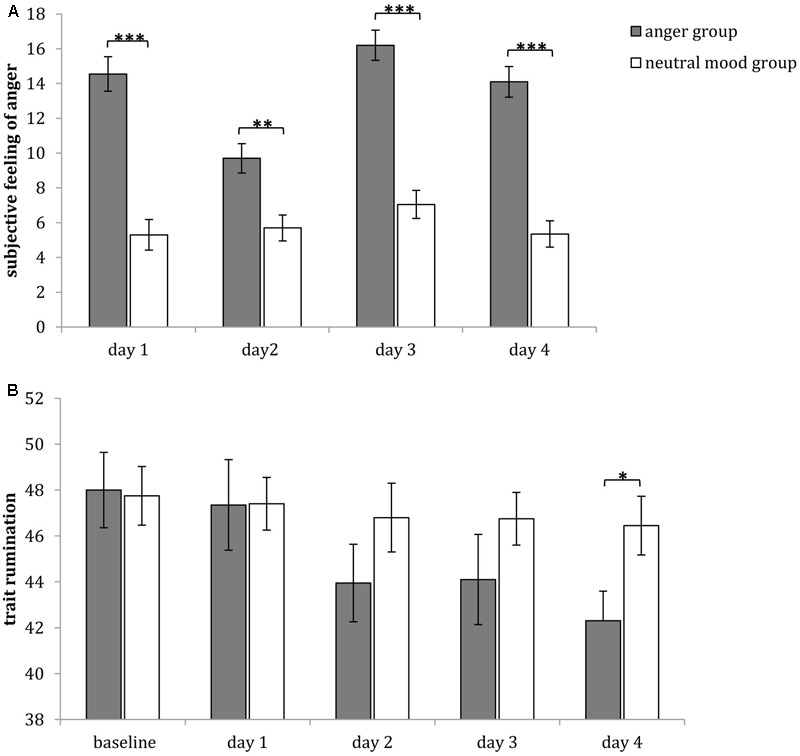
Comparisons of subjective feelings of anger **(A)** and trait rumination **(B)** after anger or neutral mood intervention each day in Study 2. Error bars (capped vertical bars) represent (–1)/(+1) *SE*. ^∗∗∗^*p* < 0.001, ^∗∗^*p* < 0.01, ^∗^*p* < 0.05.

To examine the manipulation of anger induction, we compared the angry feeling after each anger intervention and that after each neutral mood intervention. The independent-samples *t*-tests indicated that the angry feelings after anger inducing intervention was significantly higher than that after neutral mood-inducing intervention in all days [day 1: *t*_(38)_ = 3.676, *p* < 0.001, *d* = 0.734; day 2: *t*_(38)_ = 3.543, *p* < 0.01, *d* = 0.498; day 3: *t*_(38)_ = 7.718, *p* < 0.001, *d* = 0.781; day 4: *t*_(38)_ = 7.521, *p* < 0.001, *d* = 0.773] (**Figure 5A**).

#### Trait Rumination

The 5 (time: at baseline, after first intervention, after second intervention, after third intervention, after fourth intervention) × 2 (group: anger group, neutral mood group) repeated measures ANOVA of the trait rumination level indicated that the main effect of time was significant [*F*_(4,152)_ = 7.975, *p* < 0.001, η^2^ = 0.173], the main effect of group was not significant [*F*_(1,38)_ < 1, *p* > 0.05, η^2^ = 0.024], the interaction between time and group was significant [*F*_(4,152)_ = 3.327, *p* < 0.05, η^2^ = 0.081]. A simple effect analysis (sidak-adjusted) that focused on the time differences indicated that in anger group, the trait rumination level at baseline and that after first anger-inducing intervention were both significantly higher than that after second (*p*s < 0.05), third (*p*s < 0.01), and fourth intervention (*p*s < 0.001), but there were no significant differences between any two times interventions of neutral mood group (*p*s > 0.05). A simple effect analysis (sidak-adjusted) focusing on the group differences found that the trait rumination level of anger group was significantly lower than that of neutral mood group after fourth intervention (*p* < 0.05), but there were no group differences of trait rumination level between any other interventions (*p*s > 0.05), but (**Figure 5B**).

### Brief Summary of Study 2

Study 2 identified a significant downtrend of trait rumination after twice anger interventions, but no significant change was identified in the rumination tendency after any number of neutral mood interventions. Moreover, the trait rumination level after the fourth anger-inducing intervention was significantly lower than that after the fourth neutral mood-inducing intervention, implying that a certain times or intensity of anger-inducing intervention was necessary to achieve the effects of “anger counteracts (or alleviates) rumination.”

## Discussion

This study provides two key findings that supported the hypothesis of “ACR” pursuant to the MPMC theory of emotionality. First, the sad and tense feelings associated with the state rumination in the anger group were significantly lower than those in the joy group and neutral group; however, there were no group differences in the ruminative thinking frequencies, which suggests that the strategy of “anger counteracts (or alleviates) rumination” may function in reducing the negative emotion accompanied by state rumination instead of regulating ruminative thinking. Second, the trait rumination after four times anger interventions was significantly lower than that after four times neutral mood interventions, indicating that it is possible to relieve trait rumination in females using specific intensities and times of anger induction.

In Study 1, the sad, tense, and self-focus feelings in the three groups were decreased after the mood induction; this declining tendency may imply the possibility of a distraction effect, which, in general, has been recognized as an effective approach for emotional regulation by shifting one’s attention from current negative information to another unrelated activity to decrease the unpleasantness and increase healthy emotions (Nolen-Hoeksema, 1991; Donaldson et al., 2007; Yoon and Joormann, 2012). In addition, the tense feeling after joy induction was significantly less than that of the neutral induction, which may be interpreted by the effects of a positive mood or distraction in relieving rumination-related negative emotion (Nolen-Hoeksema et al., 2008; Moore, 2015). Most importantly, the sad and tense feelings of anger group were both lower than that of joy group and neutral mood group. However, the distraction or positive mood perspective could not account for the group differences among the anger induction and other two types of mood induction because the distractions that the three groups experienced were comparable in their task features (i.e., all tasks were reading evaluations from a partner), and anger was clearly not a positive emotion. In Study 2, several interventions of anger induction decreased the trait rumination (relative to the neutral mood intervention); even in the absence of a post survey regarding its persistent effects over time, this finding undoubtedly reaffirms the assumption of “anger counteracts (or alleviates) rumination” to a certain extent, which implies that anger induction may also be applicable to reduce trait rumination.

One potential mechanism for “ACR” could be considered from the perspective of how induced anger interacts with to-be-regulated rumination (Zhan et al., 2015). Specifically, we suggest that it was the interaction between anger and rumination that relieved rumination-related sadness and tension. Maladaptive ruminative in depressed individuals has been characterized by excessive DMN activation, which is proposed to underlie passive, self-relational processing (e.g., autobiographical recall, prospection) (Nolan et al., 1998; Trapnell and Campbell, 1999; Spreng et al., 2009; Hamilton et al., 2011). In contrast, anger is an intense and external-oriented emotion that involves a strong, uncomfortable and emotional response to a perceived provocation, hurt or threat, which has been shown to arouse more neural activation associated with the external attention network and executive function (e.g., conflict detection) (Antonucci et al., 2006; Boes et al., 2008; Gavita et al., 2012; Dambacher et al., 2015). It is this significant difference in the cognitive and emotional brain processes between anger and rumination that provided an opportunity for anger emotion to efficiently counteract rumination-related mental activities. Nevertheless, although the rumination-related sad and tense feelings of the anger group were significantly lower than the joy and neutral groups, the frequencies of ruminative thinking did not exhibit such difference. Thus, the rumination-related emotion (such as sadness and tension) was the aspect of rumination that could be more easily altered by anger induction; however, anger induction could not change an individual’s intrinsic tendency of rumination regarding unpleasant personal concerns. This finding implied that the ruminative state may be regulated through an emotional approach that did not involve the alteration of rumination-related cognition. In addition, it is noteworthy that Study 2 indicated an individual’s trait rumination (ruminative responses) was significantly reduced after a relatively intense anger intervention procedure. Based on this result, together with the findings obtained in Study 1 that indicated the anger induction changed the rumination-related emotion in contrast to cognition, it was possible that anger emotion interventions alleviate trait rumination through an emotional approach. Thus, the anger interventions counteracted the participants’ negative emotions caused by ruminative thinking, in turn reducing the likelihood of ruminative responses.

One interesting result in Study 1 was that the anger group exhibited less sadness and tension than the joy group. This finding was unexpected and suggests that with respect to the regulation of rumination-related emotions, such as sadness and tension, anger induction may be more efficient than the positive reward or distraction advocated by previous studies (Nolen-Hoeksema, 1991; Donaldson et al., 2007; Nolen-Hoeksema et al., 2008; Moore, 2015). Furthermore, this result appears to challenge the basic views of positive psychology, which focuses on the cultivation of an individual’s subjective well-being and emphasizes the importance of positive emotion in emotional regulation (Sheldon and King, 2001; Seligman and Csikszentmihalyi, 2014). Nevertheless, we are without intention to replace or ignore the function of positive emotion; the current findings simply suggest that as a basic negative emotion, anger may also be a potentially efficient approach to regulate rumination. It is important to realize that this study does not advocate training persons to use anger or ingraining anger into their personality to regulate rumination. In “anger counteract rumination” intervention, the anger emotion is usually not induced by a voluntary self-serve approach (such as an individual tries to intentionally anger him/herself by intentionally recalling past unpleasant experiences). These voluntary cognitive processes will, of course, require some top-down control efforts, just as other ways of cognitive regulation of emotion (such as reappraisal) does. Rather, in “anger counteract rumination” intervention, the anger emotion were usually induced by the less voluntary ways, for example, by asking individuals to passively watch anger videos or to participate in competitive game or mutual viewpoint evaluation procedure. These treatment could evoke individuals’ anger emotion but do not need them to implement intentional top-down cognitive control, and the intensity of these experimentally induced anger were also controlled in a moderate or low level relative to the one individuals encounter in their real life. So we think the application of this intervention strategy could be effortless and harmless. In spite of this, we were still very conservative in suggesting the inducing of anger as an intervention strategy for regulating rumination because anger is ultimately a type of negative emotion and it must be further investigated whether the anger intervention may result in other unwanted side effects in ruminative individuals before this approach is practically implemented.

There are several potential limitations of this study. First, the results of Study 1 did not indicate that anger could decrease state ruminative thinking frequencies. It remains unclear whether the reason was that “anger counteracts (or alleviates) rumination” directly changes the subjective feeling but not ruminative thoughts or whether it was related to an insufficient intensity of anger induction. Second, the participants in Study 2 were all females; thus, its conclusions cannot be merely extended to males. Third, this study did not compare the efficiency of “anger counteracts (or alleviates) rumination” with other strategies against rumination (e.g., reappraisal, meditation); thus, we could not assert that the anger induction has definite advantages over other strategies in relieving rumination. Fourth, maladaptive ruminative responding has been reliably associated with major depressive disorder; however, the participants in this study were not depressed. They were only individuals with relatively high ruminative tendencies in the general population. Thus, we should be cautious in generalizing the current findings to individuals with depressive disorder. Fifth, this study has examined “anger counteracts (or alleviates) rumination” by self-reported emotion experiences; future research should further consider the regulatory effects through a physiological perspective. Finally, anger is ultimately a type of negative emotion; thus, it must be investigated whether the anger intervention may result in other unwanted side effects in ruminative individuals before this approach is practically implemented.

## Conclusion

In summary, the present study demonstrated that anger induction efficiently relieved sad and tense feelings associated with state rumination; moreover, anger induction was more efficient than positive reward in the regulation of state rumination, which to some extent challenges the general viewpoint of positive psychology. In addition, several times of continuous anger interventions could decrease females’ trait rumination. These findings provided compelling evidence for the “anger counteracts (or alleviates) rumination” strategy of the MPMC theory of emotionality based on traditional Chinese medicine.

## Ethics Statement

This study was carried out in accordance with the recommendations of ‘The ethical rules of psychological experiment of human subjects, Capital Normal University’s Committee on Activities Involving Human Subjects’ with written informed consent. All subjects gave written informed consent in accordance with the Declaration of Helsinki. The protocol was approved by the ‘Capital Normal University’s Committee.’

## Author Contributions

JZ and FT performed experimental design, implement of experiment, analysis on all samples, interpreted data, wrote manuscript; JF supervised experimental design, helped to analysis all samples and interpreted data, evaluated and edit the manuscript; JL had substantial contributions to the conception or design of the work, supervised experimental design, interpreted data, edited manuscript and acted as the first corresponding author; CL supervised development of work, helped in sample collection, data interpretation, manuscript evaluation and acted as second corresponding author; JX helped to rewrite the manuscripts, supervised development of work, interpreted the data, helped to evaluate and edit the manuscript and acted as third corresponding author; MH helped to collect behavioral data, development of work, helped in data interpretation and analysing, evaluated the manuscript.

## Conflict of Interest Statement

The authors declare that the research was conducted in the absence of any commercial or financial relationships that could be construed as a potential conflict of interest.

## Footnotes

1 The original word for “thinking” in the Chinese literature is [read as si];  may indicate either the pure cognitive thinking and reasoning process that is non-pathogenic or the maladaptive repetitive thinking or ruminative thinking that is typically associated with negative emotion and has pathogenic potential. Thus,  may have different meanings in different contexts of the MPMC theory. The implication of maladaptive “thinking” in the MPMC theory of emotionality includes not only ruminative thought *per se* but also the negative, depression like emotion associated with it (Du, 2000, 2005; Jin, 2007). Therefore, in specific contexts, particularly the context discussed in this study,  indicates the ruminative or repetitive thinking that is closely related to rumination in modern psychology, which is defined as a pattern of repetitive self-focus and recursive thinking focused on negative cases or problems (e.g., unfulfilled goals or unemployment) that is always associated with the aggravation of negative mood states (e.g., sadness, tension, and self-focus) and has been shown to increase one’s vulnerability to developing or exacerbating depression (Nolen-Hoeksema, 1991; Cooney et al., 2010; Roberts et al., 2013).

## Supplementary Material

FIGURE S1 The spss output of the correlation analysis of ruminative emotion and thinking.

## References

[B1] Amodio D. M. Zinner L. R. Harmon-Jones E. (2007). “Social psychological methods of emotion elicitation,” in *Handbook of Emotion Elicitation and Assessment* eds Coan J. A. Allen J. J. B. (New York, NY: Oxford University Press) 91–105.

[B2] Antonucci A. S. Gansler D. A. Tan S. Bhadelia R. Patz S. Fulwiler C. (2006). Orbitofrontal correlates of aggression and impulsivity in psychiatric patients. *Psychiatry Res.* 147 213–220. 10.1016/j.pscychresns.2005.05.016 16952446

[B3] Behar E. Zuellig A. R. Borkovec T. (2005). Thought and imaginal activity during worry and trauma recall. *Behav. Ther.* 36 157–168. 10.1016/S0005-7894(05)80064-4

[B4] Boes A. D. Tranel D. Anderson S. W. Nopoulos P. (2008). Right anterior cingulate: a neuroanatomical correlate of aggression and defiance in boys. *Behav. Neurosci.* 122:677. 10.1037/0735-7044.122.3.677 18513137 PMC2410031

[B5] Bushman B. J. (2002). Does venting anger feed or extinguish the flame? Catharsis, rumination, distraction, anger, and aggressive responding. *Pers. Soc. Psychol. Bull.* 28 724–731. 10.1177/0146167202289002

[B6] Bushman B. J. Baumeister R. F. Phillips C. M. (2001). Do people aggress to improve their mood? Catharsis beliefs, affect regulation opportunity, and aggressive responding. *J. Pers. Soc. Psychol.* 81 17–32. 10.1037/0022-3514.81.1.17 11474722

[B7] Bushman B. J. Baumeister R. F. Stack A. D. (1999). Catharsis, aggression, and persuasive influence: self-fulfilling or self-defeating prophecies? *J. Pers. Soc. Psychol.* 76 367–376. 10.1037/0022-3514.76.3.367 10101875

[B8] Cannon W. B. (1915). “Alternative satisfactions for the fighting emotions,” in *Bodily Changes in Pain, Hunger, Fear and Rage: An Account of Recent Researches into the Function of Emotional Excitement* ed. Cannon W. B. (New York City, NY: D. Appleton & Company) 285–301.

[B9] Conway M. Csank P. A. Holm S. L. Blake C. K. (2000). On assessing individual differences in rumination on sadness. *J. Pers. Assess.* 75 404–425. 10.1207/S15327752JPA7503_04 11117154

[B10] Cooney R. E. Joormann J. Eugène F. Dennis E. L. Gotlib I. H. (2010). Neural correlates of rumination in depression. *Cogn. Affect. Behav. Neurosci.* 10 470–478. 10.3758/CABN.10.4.470 21098808 PMC4476645

[B11] Dambacher F. Sack A. T. Lobbestael J. Arntz A. Brugman S. Schuhmann T. (2015). Out of control: evidence for anterior insula involvement in motor impulsivity and reactive aggression. *Soc. Cogn. Affect. Neurosci.* 10 508–516. 10.1093/scan/nsu077 24837479 PMC4381232

[B12] Donaldson C. Lam D. Mathews A. (2007). Rumination and attention in major depression. *Behav. Res. Ther.* 45 2664–2678. 10.1016/j.brat.2007.07.002 17692819

[B13] Du W. D. (2000). The meaning of the word Si and its clinical significance. *J. Nanjing Univ. TCM* 1 100–102.

[B14] Du W. D. (2005). *Psychology of Traditional Chinese Medicine.* Beijing: China Medical Science Press.

[B15] Gavita O. A. Capris D. Bolno J. David D. (2012). Anterior cingulate cortex findings in child disruptive behavior disorders: a meta-analysis. *Aggress. Violent Behav.* 17 507–513. 10.1016/j.avb.2012.07.002

[B16] Giancola P. R. Parrott D. J. (2008). Further evidence for the validity of the Taylor aggression paradigm. *Aggress. Behav.* 34 214–229. 10.1002/ab.20235 17894385

[B17] Gortner E.-M. Rude S. S. Pennebaker J. W. (2006). Benefits of expressive writing in lowering rumination and depressive symptoms. *Behav. Ther.* 37 292–303. 10.1016/j.beth.2006.01.004 16942980

[B18] Hamilton J. P. Furman D. J. Chang C. Thomason M. E. Dennis E. Gotlib I. H. (2011). Default-mode and task-positive network activity in major depressive disorder: implications for adaptive and maladaptive rumination. *Biol. Psychiatry* 70 327–333. 10.1016/j.biopsych.2011.02.003 21459364 PMC3144981

[B19] Han X. Yang H. F. (2009). Chinese version of Nolen-Hoeksema ruminative responses scale (RRS) used in 912 college students: reliability and validity. *Chin. J. Clin. Psychol.* 17 550–551.

[B20] Huang L. Yang T. Ji Z. (2003). Applicability of the positive and negative affect scale in Chinese. *Chin. Ment. Health J.* 17 54–56.

[B21] Jin G. L. (2007). The history and concept of emotion in traditional Chinese medicine. *J. Beijing Univ. Tradit. Chin. Med.* 30 514–516. 10.1007/s11013-012-9290-y 23315392 PMC3586067

[B22] Keng S.-L. Smoski M. J. Robins C. J. (2016). Effects of mindful acceptance and reappraisal training on maladaptive beliefs about rumination. *Mindfulness* 7 493–503. 10.1007/s12671-015-0480-x

[B23] Kingston T. Dooley B. Bates A. Lawlor E. Malone K. (2007). Mindfulness-based cognitive therapy for residual depressive symptoms. *Psychol. Psychother.* 80 193–203. 10.1348/147608306X116016 17535594

[B24] Li S. T. Wang G. Y. Weng N. Zeng L. Wang M. Q. (2007). The application of the method of counteraction regulating. *Chin. J. Integr. Tradit. Western Med.* 16 3613–3614.

[B25] Lv B. W. Bi Y. (2002). *Lushi Chunqiu* Vol. 582 Shanghai: Zhonghua Book Company.

[B26] Lyubomirsky S. Nolen-Hoeksema S. (1995). Effects of self-focused rumination on negative thinking and interpersonal problem solving. *J. Pers. Soc. Psychol.* 69 176–190. 10.1037/0022-3514.69.1.176 7643299

[B27] Martin L. L. Shrira I. Startup H. M. (2004). “Rumination as a function of goal progress, stop rules, and cerebral lateralization,” in *Depressive Rumination: Nature Theory and Treatment* (Chichester: John Wiley & Sons) 153–176.

[B28] Martin L. L. Tesser A. (1996). Some ruminative thoughts. *Adv. Soc. Cogn.* 9 1–47.

[B29] McLaughlin K. A. Nolen-Hoeksema S. (2011). Rumination as a transdiagnostic factor in depression and anxiety. *Behav. Res. Ther.* 49 186–193. 10.1016/j.brat.2010.12.006 21238951 PMC3042543

[B30] Moore K. (2015). Rumination and self-destructive thoughts in people with depression. *Behav. Sci. Undergrad. J.* 2 5–12.

[B31] Nolan S. A. Roberts J. E. Gotlib I. H. (1998). Neuroticism and ruminative response style as predictors of change in depressive symptomatology. *Cogn. Ther. Res.* 22 445–455. 10.1023/A:1018769531641

[B32] Nolen-Hoeksema S. (1991). Responses to depression and their effects on the duration of depressive episodes. *J. Abnorm. Psychol.* 100 569–582. 10.1037/0021-843X.100.4.569 1757671

[B33] Nolen-Hoeksema S. Morrow J. (1991). A prospective study of depression and posttraumatic stress symptoms after a natural disaster: the 1989 Loma Prieta Earthquake. *J. Pers. Soc. Psychol.* 61 115–121. 10.1037/0022-3514.61.1.115 1890582

[B34] Nolen-Hoeksema S. Wisco B. E. Lyubomirsky S. (2008). Rethinking rumination. *Perspect. Psychol. Sci.* 3 400–424. 10.1111/j.1745-6924.2008.00088.x 26158958

[B35] Rabasco A. Zakon M. Li M. (2015). “Regulating anger experience: the benefits of distraction over rumination, acceptance, and reappraisal,” in *Proceedings of the 2015 SAS Annual Conference: Society for Affective Science* Los Angeles, CA.

[B36] Raio C. M. Orederu T. A. Palazzolo L. Shurick A. A. Phelps E. A. (2013). Cognitive emotion regulation fails the stress test. *Proc. Natl. Acad. Sci. U.S.A.* 110 15139–15144. 10.1073/pnas.1305706110 23980142 PMC3773739

[B37] Ray R. D. Wilhelm F. H. Gross J. J. (2008). All in the mind’s eye? Anger rumination and reappraisal. *J. Pers. Soc. Psychol.* 94 133–145. 10.1037/0022-3514.94.1.133 18179323

[B38] Roberts H. Watkins E. R. Wills A. J. (2013). Cueing an unresolved personal goal causes persistent ruminative self-focus: an experimental evaluation of control theories of rumination. *J. Behav. Ther. Exp. Psychiatry* 44 449–455. 10.1016/j.jbtep.2013.05.004 23810947

[B39] Robertson I. H. Manly T. Andrade J. Baddeley B. T. Yiend J. (1997). Oops!’: performance correlates of everyday attentional failures in traumatic brain injured and normal subjects. *Neuropsychologia* 35 747–758. 10.1016/S0028-3932(97)00015-8 9204482

[B40] Roley M. E. Claycomb M. A. Contractor A. A. Dranger P. Armour C. Elhai J. D. (2015). The relationship between rumination, PTSD, and depression symptoms. *J. Affect. Disord.* 180 116–121. 10.1016/j.jad.2015.04.006 25898331

[B41] Rusting C. L. Nolen-Hoeksema S. (1998). Regulating responses to anger: effects of rumination and distraction on angry mood. *J. Pers. Soc. Psychol.* 74 790–803. 10.1037/0022-3514.74.3.790 9523420

[B42] Segal Z. V. Williams J. M. G. Teasdale J. D. (2012). *Mindfulness-based Cognitive Therapy for Depression.* New York City, NY: Guilford Press.

[B43] Seligman M. E. Csikszentmihalyi M. (2014). *Positive Psychology: An Introduction.* Berlin: Springer. 10.1037//0003-066x.55.1.5 11392865

[B44] Sheldon K. M. King L. (2001). Why positive psychology is necessary. *Am. Psychol.* 56 216–217. 10.1037/0003-066X.56.3.216 11315247

[B45] Sloan D. M. Marx B. P. Epstein E. M. Dobbs J. L. (2008). Expressive writing buffers against maladaptive rumination. *Emotion* 8 302–306. 10.1037/1528-3542.8.2.302 18410204

[B46] Spreng R. N. Mar R. A. Kim A. S. (2009). The common neural basis of autobiographical memory, prospection, navigation, theory of mind, and the default mode: a quantitative meta-analysis. *J. Cogn. Neurosci.* 21 489–510. 10.1162/jocn.2008.21029 18510452

[B47] Taylor S. P. (1967). Aggressive behavior and physiological arousal as a function of provocation and the tendency to inhibit aggression1. *J. Pers.* 35 297–310. 10.1111/j.1467-6494.1967.tb01430.x 6059850

[B48] Trapnell P. D. Campbell J. D. (1999). Private self-consciousness and the five-factor model of personality: distinguishing rumination from reflection. *J. Pers. Soc. Psychol.* 76 284–304. 10.1037/0022-3514.76.2.284 10074710

[B49] Wager T. D. Kang J. Johnson T. D. Nichols T. E. Satpute A. B. Barrett L. F. (2015). A Bayesian model of category-specific emotional brain responses. *PLOS Comput. Biol.* 11:e1004066. 10.1371/journal.pcbi.1004066 25853490 PMC4390279

[B50] Wang H. T. (1999). *Inner Canon of the Yellow Emperor.* Beijing: New World Press.

[B51] Wang M. Q. (2007). *Modern Chinese Medicine Psychology.* Beijing: Chinese Traditional Chinese Medicine Press.

[B52] Watkins E. R. Nolen-Hoeksema S. (2014). A habit-goal framework of depressive rumination. *J. Abnorm. Psychol.* 123 24–34. 10.1037/a0035540 24661156

[B53] Watson D. Tellegen A. (1985). Toward a consensual structure of mood. *Psychol. Bull.* 98 219–235. 10.1037/0033-2909.98.2.219 3901060

[B54] Xu P. Huang Y. Luo Y. (2010). Establishment and assessment of native Chinese affective video system. *Chin. Ment. Health J.* 24 551–554.

[B55] Yoon K. L. Joormann J. (2012). Is timing everything? Sequential effects of rumination and distraction on interpersonal problem solving. *Cogn. Ther. Res.* 36 165–172. 10.1007/s10608-010-9330-2

[B56] Zetsche U. D’Avanzato C. Joormann J. (2012). Depression and rumination: relation to components of inhibition. *Cogn. Emot.* 26 758–767. 10.1080/02699931.2011.613919 21970297

[B57] Zhan J. Ren J. Fan J. Luo J. (2015). Distinctive effects of fear and sadness induction on anger and aggressive behavior. *Front. Psychol.* 6:725. 10.3389/fpsyg.2015.00725 26124725 PMC4467173

[B58] Zhan J. Wu X. Fan J. Guo J. Zhou J. Ren J. (2017). Regulating anger under stress via cognitive reappraisal and sadness. *Front. Psychol.* 8:1372. 10.3389/fpsyg.2017.01372 28855881 PMC5557741

[B59] Zhang Y. (1991). The measurement of experimentally induced affects. *Acta Psychol. Sin.* 1 99–99.

[B60] Zuckerman M. Lubin B. (1985). *Manual for the Multiple Affect Adjective Check List.* San Diego, CA: Educational and Industrial Testing Service.

