# Supplementary figures and images for: Regulating Rumination by Anger: Evidence for the Mutual Promotion and Counteraction (MPMC) Theory of Emotionality
Source: Front Psychol. 2017 Dec 1;8:1871. doi: 10.3389/fpsyg.2017.01871 (PMC5716968; doi:10.3389/fpsyg.2017.01871)

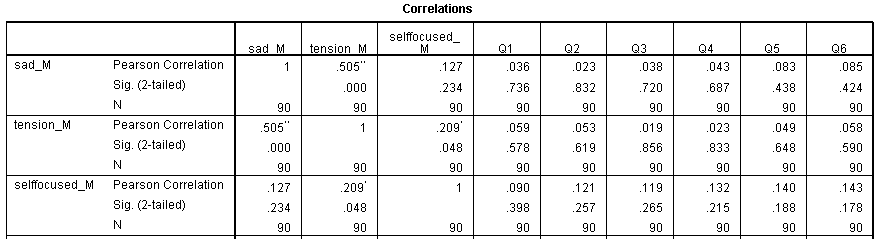

Supplement: FIGURE S1 — The spss output of the correlation analysis of ruminative emotion and thinking. [file Image_1.PNG]
